# Externalizing Problem Behaviors Among Chinese Early Adolescents in Poverty: Profiles and Longitudinal Change

**DOI:** 10.1002/pchj.70037

**Published:** 2025-07-15

**Authors:** Miqi Li, Zhihang Wang, Zhihua Li

**Affiliations:** ^1^ College of Education Hunan University of Science & Technology Xiangtan China; ^2^ College of Public Administration and Law Hunan Agricultural University Changsha China

**Keywords:** early adolescence, externalizing problem behaviors, family factors, latent profile analysis, latent transition analysis, poverty

## Abstract

Children's externalizing problem behavior is one of the most explored topics among parents, educators, and research scholars. The purpose of this study is to examine the developmental changes of externalizing problem behavior in the early years of poor children and adolescents and the influence of family factors such as family functioning and parental marital quality on the developmental changes. Seven hundred and seventy‐eight early adolescents (M_age_ = 13.7, SD = 2.53) from poor families were studied longitudinally for 14 months. The results showed that three potential characteristics of externalizing problem behavior patterns were identified through Latent Profile Analysis (LPA): *well‐adjusted group, attention disorder group, and conduct problem group*. Latent Transition Analysis (LTA) revealed a tendency for the *conduct problem group* to transition to the *well‐adjusted group* over two traces (OR = 0.40). There were gender differences in the results: boys in the *conduct problem group* were more likely to transition to the *well‐adjusted group* (OR = 0.55), while girls in the *attention disorder group* were more likely to transition to the *well‐adjusted group* (OR = 2.63). Research has found that a supportive family environment is a positive factor in mitigating externalizing problem behaviors of the early adolescents in their transition to adolescence.

## 1 Introduction

According to the report of Global Multidimensional Poverty Index in 2023 published by the United Nations Development Program, 1.1 billion people in the world are in a “state of multidimensional poverty”, of which children and adolescents make up the largest portion: about 556 million under the age of 18. Children in poverty experience deprivation of resources needed to survive and develop, and most often, they cannot bring out their potential or participate in the society as full and equal members (United Nations International Children's Emergency Fund [UNICEF] 2005). Research has shown that the adverse circumstances as a result of poverty can seriously hinder their physical and mental development. For example, children in poor families are more likely than those in non‐poor families to have problems, such as physical developmental delay (Buckley et al. 2019), inadequate cognitive development (Racz et al. 2017), serious emotional internalization disorder (Raver et al. 2016), and obvious externalizing problem behaviors (Li et al. 1995). Externalizing problem behavior is the most intuitive and prominent problem in children's development, and also the most difficult problem in children's education work (Guan 2018). Thus this study focuses on the externalizing problem behaviors of a sample of early adolescents in poverty in China.

### 1.1 Externalizing Problem Behaviors and Patterns of Differentiation Among Adolescents in Poverty

Externalizing problem behaviors refer to a group of symptoms, such as attention deficit, hyperactivity, resistance bias behaviors, and aggression. These behaviors mainly present themselves in two forms: conduct problems and hyperactivity (McGee et al. 1992). Previous research has shown that children of low socioeconomic families tend to have more externalizing problem behaviors, such as smoking, drinking, and aggression (Haushofer and Fehr 2014; Letourneau et al. 2013). Eccles et al. (1993) suggest that according to stage‐environment fit theory, individuals are less likely to do well and be motivated if the environment in which they live does not meet their psychological needs. Living in poverty places a great deal of stress on parents' lives, which in turn can affect their ability to meet their children's psychological needs and lead to behavioral problems.

Research has also shown that externalizing problem behaviors of the children in poverty were influenced by, or related to, a number of factors, among which age has been shown to be an important factor (Miner and Clarke‐Stewart 2008). From late childhood to early adolescence, the externalizing problem behaviors would have an “explosive” growth (Atherton et al. 2018; Costello et al. 2011). Moffitt discussed that externalizing problem behaviors in adolescence usually have the characteristics of “beginning in adolescence,” “only appearing in adolescence,” and “being non‐aggressive or antisocial” (Moffitt 1993). In other words, early adolescence is the period when children may undergo abrupt changes in behaviors (Chung and Gale 2009). According to the developmental readiness hypothesis (Mendle et al. 2007), the adolescent transition is a critical period of change and social processes for children. Children in early adolescence are emotionally and physically unprepared for the biological and environmental changes of adolescence and are therefore at risk of internalizing and externalizing problematic behaviors (Pinquart 2017). Externalizing problem behaviors in adolescence are strongly associated with multiple maladaptive outcomes in their current and over time, such as lower academic achievement, peer rejection, substance addiction, and delinquent behaviors (Janssens et al. 2015; Lewis et al. 2017; Reef et al. 2011). All these suggest that better understanding of the externalizing problem behavior patterns at the time of early adolescence could be critical.

Therefore, this study focused on the period of early adolescence in order to have a better understanding of the development of externalizing problem behaviors among early adolescents in poverty.

### 1.2 The Influence of Family Factors on Adolescents' Externalizing Problems

As the earliest microsystem that individuals are exposed to, adolescents' early externalizing problem behaviors are influenced by family factors. According to bio‐ecological theory, as children grow older, their sense of self and independence increases and their ability to think dialectically and logically develops further, making adolescents increasingly influenced by their peers, relatives, and youth groups, and gradually changing the mode of interaction with their parents from parent‐dominated to equal communication. According to the positive adolescent development concept, a variety of positive factors such as high self‐control, social competence, parental monitoring, family support, emotional support from teachers, a positive school climate, and neighborhood cohesion can significantly reduce the prevalence of externalizing problem behaviors among adolescents. A good family environment can prevent or reduce externalizing problem behaviors, which can effectively reduce the incidence of adolescents' externalizing problem behaviors on the one hand, help adolescents develop other positive behaviors (e.g., pro‐social behaviors), and replace undesirable behavioral problems by promoting the continuous development of positive behaviors, thus reducing the incidence of externalizing problem behaviors to a greater extent on the other hand (Burk and Laursen 2010; Chang et al. 2019).

Previous studies have shown that among family‐related factors, parental education, parental marital quality, and family functioning have significant effects on adolescent externalizing behavior problems. Wang and colleagues showed that low socioeconomic status parents tend to be harsh disciplinarians, which in turn leads to externalizing problems in their children (Wang and Liu 2014, 2018). In addition to family economic status, parental marital quality also directly and positively predicts adolescent externalizing problems (Wu et al. 2017). Furthermore, Ettekal et al. (2019) showed that family stressful events positively predicted externalizing problems in children. Individuals from families with poor family functioning are at higher risk of substance use and developing heavy/problematic use (Hummel et al. 2013). Conversely, higher maternal warmth was negatively associated with initial levels of externalizing behavior problems in adolescents, and higher paternal warmth was indirectly positively associated with initial levels of externalizing behavior problems and was associated with a downward trend in externalizing problem behaviors (Pereyra et al. 2019).

Contextual amplification theory suggests that a poor family environment interacts with adolescent transitions to increase the risk of externalizing problems (Ge and Natsuaki 2009). Therefore, this study hypothesized that a supportive family environment (parents' educational background, family functioning, and parents' marital quality) has a positive effect on the developmental changes in early externalizing problem behaviors of poor adolescents in 14 months.

### 1.3 Gaps in the Research Literature

Much progress has been made in understanding the relevant issues related to externalizing problem behaviors in children and adolescents. Past research in this area, however, had some limitations. First, the total summed score of a measure of externalizing problem behaviors, or factor scores on a measure of externalizing problem behaviors, was usually used for classifying or characterizing individuals in terms of their externalizing problem behaviors. Individuals classified into the same subcategory (e.g., those with similar total scores) may have very different response patterns on the items of the measure of externalizing problem behaviors, and such different response patterns could suggest substantial differences in the subcategory's population (Petersen et al. 2015). In other words, the use of total scores could often camouflage meaningful differences (e.g., different profiles of externalizing problem behaviors) among the individuals grouped into the same subcategory. In this study, we aimed at understanding the externalizing problem behavior patterns based on response patterns as exhibited at the item level, instead of relying on the sum score or total score of a measure. This approach would allow us to explore the possibility that there could be different profiles of externalizing problem behaviors that would not be obvious if the total score of a measure was the focus, as in many previous studies.

Second, there is a lack of understanding about how externalizing problem behaviors would change over time, especially in early adolescence. In adolescence, which is full of changes, adolescents who previously belonged to one adaptation subgroup may shift to another subgroup due to changes in some of their adaptation indicators. Previous research in this area primarily focused on the relationships between externalizing problem behaviors and other factors (Chen et al. 2009; Costello et al. 2011), but there has been relatively little research on changes, or lack thereof, in externalizing problem behaviors over time.

### 1.4 The Present Study

In this study, we were interested in understanding several issues related to possible longitudinal changes of externalizing problem behaviors, such as possible dynamic changes over time in the externalizing problem behaviors in early adolescence (Di Giunta et al. 2018), stability differences among different patterns of externalizing problem behaviors (e.g., non‐aggressive conduct problems versus aggressive externalizing problem behaviors; Donker et al. 2003; Qu et al. 2018), possible gender differences in the stability of non‐aggressive behavior disorder (Playford et al. 2017; Womack et al. 2019).

In this study, we would: (1) explore possible latent (i.e., unobserved) patterns (profiles) of externalizing problem behaviors in a sample of early adolescents in poverty; (2) examine the change of such externalizing problem behavior patterns over a two‐year period of time in early adolescence; and (3) examine how some family factors influenced such longitudinal change of the externalizing problem behaviors. For the first goal, latent profile analysis (LPA; He and Fan 2019) was used to identify different patterns of externalizing problem behaviors based on the response patterns of the participants on the items measuring externalizing problem behaviors. For the remaining two goals, latent transition analysis (LTA; Bray and Dziak 2018) was used to study the change of the externalizing problem behavior patterns of the early adolescents in poverty, and to study how some family factors (e.g., parents' education, family function, parents' marriage quality) influenced such behavior changes. Both LPA and LTA are individual‐centered data analysis methods, with the latter focusing on longitudinal development and change, and estimating the transition probabilities of individuals from one profile to another (Moore et al. 2019). This study hypothesized that (1) externalizing behavior problems in early adolescence produce shifts over time, and (2) potential categories of externalizing behavior problems are influenced by gender as well as family factors; individuals with high levels of parental education, well‐functioning families, and high‐quality parental marriages may have fewer externalizing behavior problems or be more likely to shift to well‐behaved groups over time.

## 2 Methods

### 2.1 Participants

The data for the current work was from a longitudinal study about the behavioral problems of children from poor families. Based on school registration records of poor families certified by the local governments, we contacted 34 junior middle schools in a province in central China for conducting a longitudinal survey among children and their parents from poor families whose family income was below the local income standard (per capita net income in Hunan Province in 2017 was 3026 yuan). After introducing the study to the students and their parents, from a total of 832 eligible students, 778 students and their parents were willing to participate, and informed consents were obtained from these children and their parents.

In the study, there were two waves of data collection. The first wave was in August 2018, and questionnaires from 778 students were collected. This wave of data collection involved one child and one of his/her parents. The first wave included 310 boys (39.8%) and 468 girls (60.2%), 198 students from poverty families in urban areas and 580 from poverty families in rural areas. Of the 778 children, 203 were in Grade 7, 340 in Grade 8, and 235 in Grade 9. The average age of the children was 13.7 (SD = 2.53), and the average age of their fathers was 41.09 (SD = 6.21) and that of mothers was 37.87 (SD = 6.07). Fourteen months later, we collected a second wave of data. Twenty‐six of these subjects were lost due to reasons such as transfer and graduation. The second wave included 300 boys (39.8%) and 452 girls (60.2%), with 196 students from poverty families in urban areas and 556 from poverty families in rural areas. Of the 752 children, 189 were in Grade 7, 332 in Grade 8, and 231 in Grade 9. The average age of the children was 14.8 (SD = 2.47).

The results of *chi‐square* and independent‐sample t‐tests showed that there was no statistical difference in all the key variables (gender ratio, age, father/mother education background, family marriage quality, family function, externalizing problem behaviors) between those remaining in the second wave of data collection (*N* = 752) and the 26 students lost in the second wave of data collection, suggesting that the sample attrition from the first to second wave data collection was very unlikely to have introduced any potential bias in the final sample to be used in the subsequent data analyses.

### 2.2 Procedures

Researchers organized a questionnaire for the students to administer during study sessions and for the students to take the parental questionnaire home and invite the parents to fill it out and bring it back to school the next day. Informed consent was obtained from these children and their parents for this study. Upon completion of both waves of data collection, each student received a gift valued at 50 Chinese yuan (around 7 US dollars) as a token of appreciation. All data was kept confidential and only the researchers in the project could access the data. The longitudinal survey was carried out in accordance with the latest version of the Declaration of Helsinki and approved by the Ethics Committees of Hunan University of Science and Technology.

### 2.3 Measures

#### 2.3.1 Demographic Variables

The students reported information about gender (dummy coded: boy = 1, girl = 2), age, and grade. Parents reported their age, educational background, and per capita monthly income of the family. Based on Buehler and Gerald's method (Buehler and Gerard 2013), the educational level of the father and mother was measured separately (illiteracy = 1, elementary school = 2, junior high school = 3, senior high school or technical secondary school = 4, junior college = 5, bachelor degree = 6, master's degree or above = 7), and the average value of the educational level of the parents was used in later analyses.

#### 2.3.2 Externalizing Problem Behaviors

*The self‐report version of* the *Strengths and Difficulties Questionnaire* (SDQ) of Children by Goodman (Goodman et al. 1998) was used in this study. This instrument contains 25 items and evaluates five dimensions: emotional symptoms, conduct problems, hyperactive disorder, peer interaction problems, and prosocial behavior. In this study, we used two (conduct problems and hyperactivity disorder; hyperactivity disorder is used to measure attention disorder problems) of the five sub‐measures to measure the degree of externalizing problem behaviors of children (Roman et al. 2016). Each of these two sub‐measures has five items, and each item is scored on a 3‐point scale (0–2), with a higher score indicating a higher level of externalizing problem behaviors. The Chinese version of SDQ showed good reliability in the Chinese population (Yao et al. 2009). In this study, the two sub‐measures had Cronbach's α coefficient of 0.78 and 0.81, respectively. The Appendix presents the items on these two sub‐measures.

#### 2.3.3 Parental Marriage Quality

In the first wave of data collection, parents responded to items related to their marriage quality. The measure used was the subscale of marriage and family quality in the *Generic Quality of Life Inventory‐74* (GQOLI‐74) (Li et al. 1995), which showed good applicability in the Chinese population. The measure included items assessing marital relationship status, frequency of marital communication, satisfaction with marital status, amount of shared housework, and satisfaction with household responsibilities (a representative item on this scale was: “In the past week, when one or both spouses are distressed, they often have mutual,” choose from 1 (never communicate) to 5 (communicate with each other)). The total score of the subscale was used as the indicator of the family marriage quality, with higher score indicating better marriage quality. In this study, the Cronbach's α coefficient for this measure of family marriage quality was 0.86.

#### 2.3.4 Family Function

The *Family APGAR Index Questionnaire* (Takenaka and Ban 2016) was used for measuring family function. The questionnaire included five items for adaptation, cooperation, growth, emotion, and intimacy (a representative item on this scale was: “I can get satisfactory help from my family when I am in trouble”), which were answered by parents. The 3‐point scoring method of 0–2 was used. The sum of the five items indicates family function, with higher score indicating better family function (total score of 7–10 for well‐functioning, 4–6 for moderate disorder in the family function, and 0–3 indicating serious disorder in the family function). In this study, Cronbach's *α* coefficient of the questionnaire was 0.81.

### 2.4 Data Analysis

PROCLTA (Lanza et al. 2013) was used in SAS 9.4 for the Latent Transition Analysis. Latent transition analysis requires a series of progressive analytical steps. First, latent profile analysis (LPA) was conducted to identify latent (i.e., unobserved) groups with statistically distinguishable patterns of externalizing problem behaviors (He and Fan 2019). Referring to previous studies, we estimated models with 1–4 emotional symptoms categories. Starting with the initial model (assuming that only one category exists for all samples), the number of categories in the model is gradually increased until the model that fits the data best is found. Once such patterns (profiles) were appropriately identified for the two time points (i.e., at both time points, the similar behavior patterns were identified), analysis was further conducted to examine the status of change and transition probability of the individuals across the different “profiles” of externalizing problem behaviors from T1 to T2. Finally, the family factors were incorporated into the LTA model as covariates to examine the influence of these family factors on the membership status in different profiles and on the transition across the profiles over time. For technical details (i.e., identification of latent profiles, model fit assessment, etc.) of LPA and LTA, readers are referred to more technical sources that are readily available (e.g., list of Bray and Dziak 2018; He and Fan 2019; Moore et al. 2019).

## 3 Results

### 3.1 Descriptive Statistics

We measured the mean level and the standard deviations of externalizing problem behaviors (M_T1_ = 5.446, SE_T1_ = 3.064; M_T2_ = 6.123, SE_T2_ = 3.257), family function (M = 7.20, SE = 1.919) and parental marriage quality (M = 15.031, SE = 2.260). See Table 1. The results show that the mean externalizing problem behaviors between the two surveys varied very little, which implies that the mean externalizing problem behaviors trend had a certain degree of stability. ANOVA was conducted for the 10 items ofT1 and T2 externalizing problem behaviors, and the results showed significant differences between the 10 items (*ps* < 0.01).

**TABLE 1:** Mean, standard deviation, and correlation coefficient matrix of variables (*N* = 752).

	M	SD	1	2	3	4	5
Gender^a^	—	—	—				
Parents' education level^b^	3.01	0.766	0.076*	—			
Parental marital quality	15.031	2.260	−0.108	0.147**	—		
Family function	7.20	1.919	0.088*	0.155**	0.232**	—	
T1	5.446	3.064	−0.048	−0.089*	−0.120**	−0.075*	—
T2	6.123	3.257	0.027	−0.024	−0.129**	−0.088*	0.273**

^a^ Gender (1 = boy, 2 = girl) and parents' education level.

^b^ Dummy variables.

### 3.2 Identification of Latent Profiles

Through Latent Profile Analysis (LPA), the optimal LPA model for both T1 and T2 response data on the 10 items measuring externalizing problem behaviors (conduct problems and hyperactivity disorder, each with five items) was identified. In the LPA modeling fitting, models with 1 to 4 latent profiles were extracted and their respective model fit examined. The fit indexes for both T1 and T2 data for each LPA model (models with 1 to 4 latent profiles) are shown in Table 2. According to the criteria that Lo–Mendell–Rubin (LMR) and Bootstrapped Likelihood Ratio Test (BLRT) are significant, minimal AIC, BIC, and BIC values, and the proportion of the minimum class is not less than 5%, the development trajectory of emotional symptoms can be divided into three classes. From Table 2, it can be seen that the optimal model for T1 response data was a three‐profile model, as the incremental improvement of model fit of the four‐profile model over the three‐profile model was no longer statistically significant. For T2 response data, there was a little uncertainty. The incremental improvement of model fit of the four‐profile model over the three‐profile model was still statistically significant at *α* = 0.05 level, although not at the *α* = 0.01 level as the previous incremental improvement (i.e., three‐profile model over two‐profile model, two‐profile model over one‐profile model). In addition, if the four‐profile model was selected, the fourth profile would only contain 4.01% of the total observations, lower than the 5% that is typically considered adequate in LPA analysis (e.g., Cumsille et al. 2015). After all relevant model fit information is considered, the three‐profile latent transition model was selected.

**TABLE 2:** Model fit indices of latent profile analysis (*N* = 752).

Times	No. profiles	Log (L)	AIC	BIC	aBIC	Entropy	*p*
T1	1	−7439.49	14918.97	15012.49	14948.98	1.00	< 0.01
	2	−6809.17	13680.35	13825.30	13726.86	1.00	< 0.01
	**3**	**−6605.77**	**13295.55**	**13491.93**	**13358.56**	**0.88**	**< 0.01**
	4	−6350.98	12807.96	13055.78	12887.47	0.81	> 0.05
T2	1	−7165.63	14371.26	14463.15	14399.65	1.00	< 0.01
	2	−6572.13	13206.26	13348.68	13250.25	1.00	< 0.01
	**3**	**−6327.13**	**12738.26**	**12931.23**	**12797.86**	**0.88**	**< 0.01**
	4	−6243.16	12592.31	12835.81	12667.52	0.88	< 0.05

*Note*: Bold indicates final solution.

### 3.3 Naming of the Three Profile Groups

**FIGURE 1:**
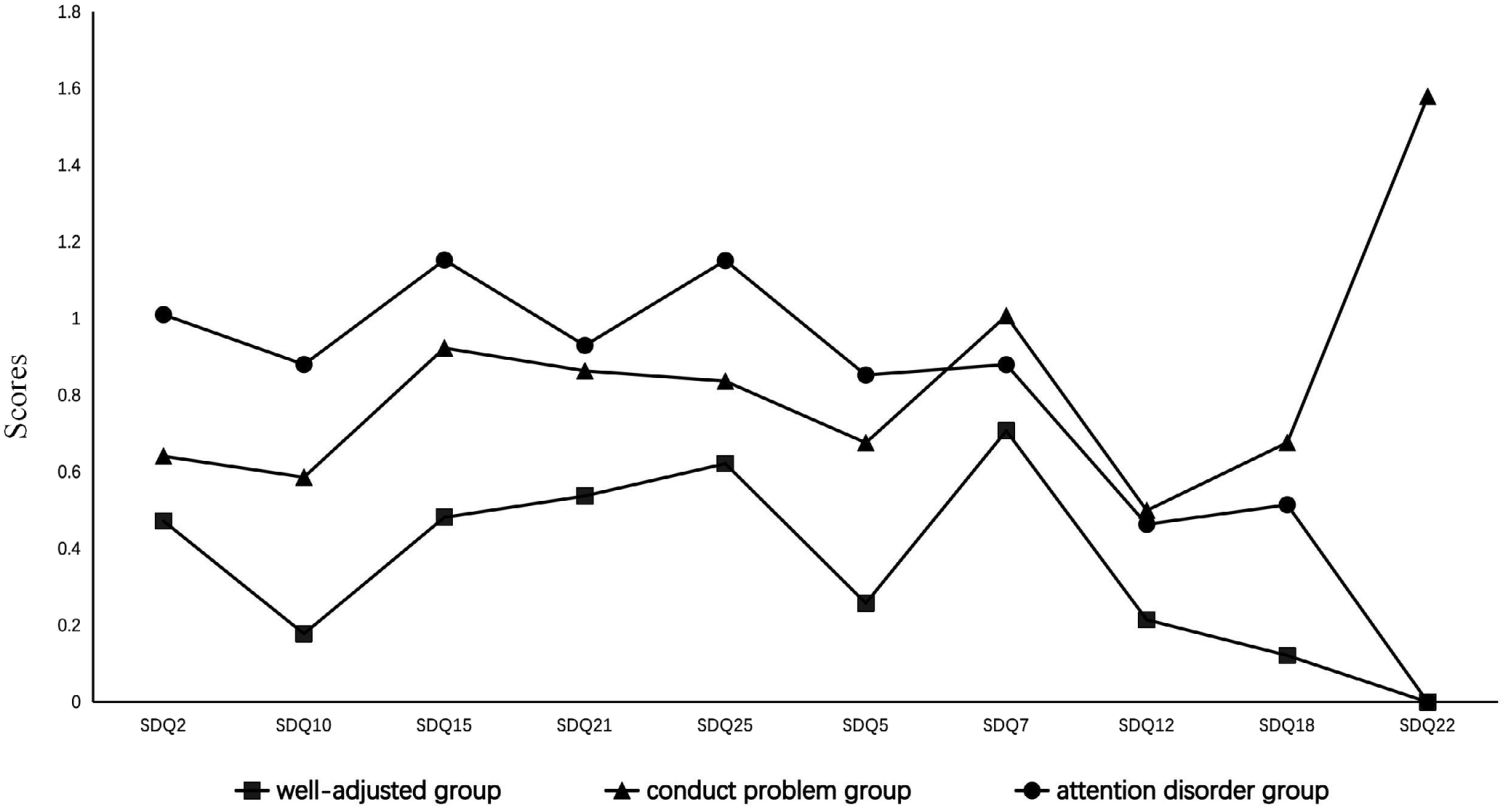
Latent profile analysis (LPA) classification results of externalizing problem behaviors at T1.

**FIGURE 2:**
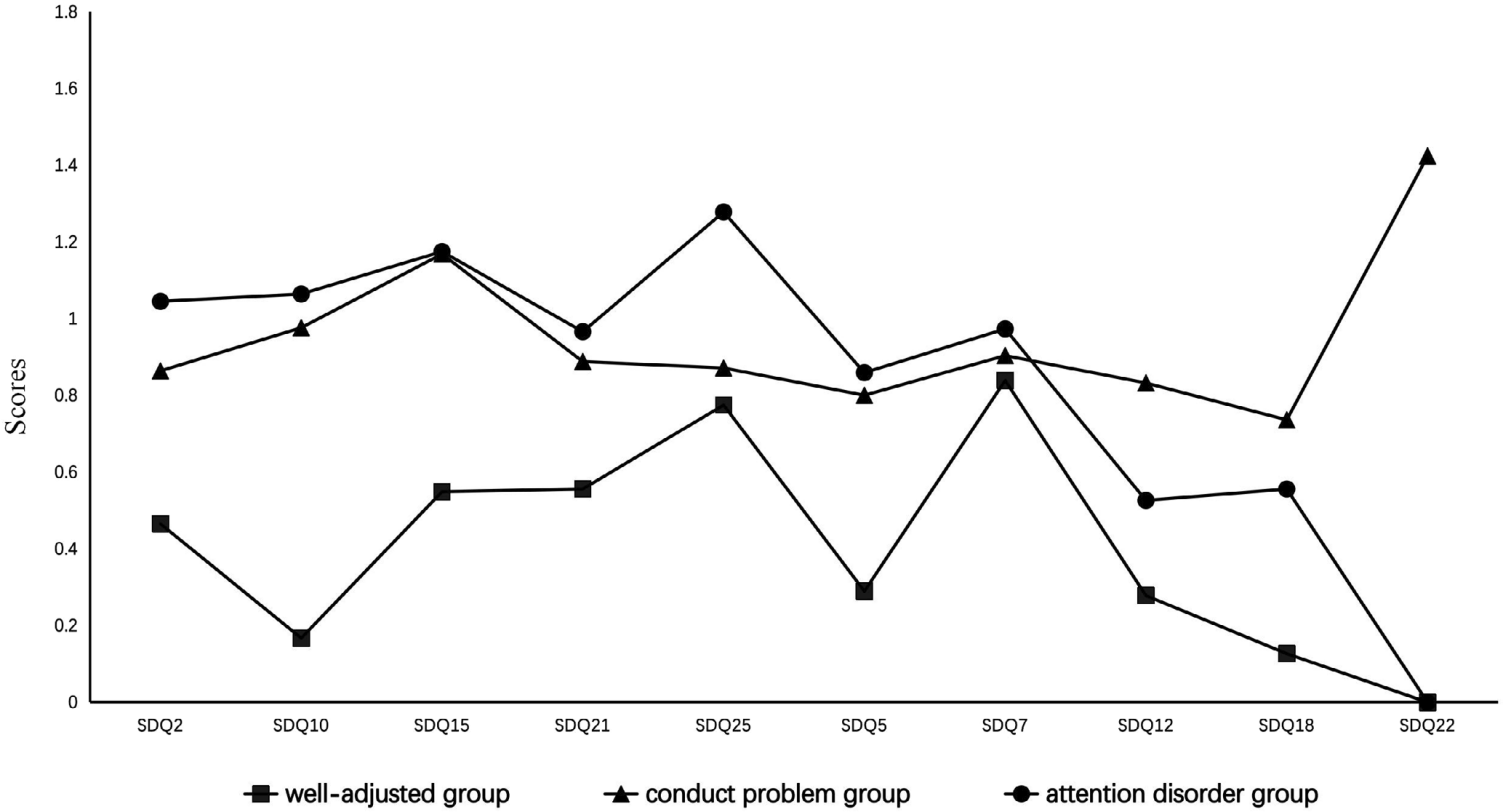
Latent profile analysis (LPA) classification results of externalizing problem behaviors at T2.

The three‐profile LPA model for T1 and T2 was analyzed and examined. The three latent profiles (or unobserved groups) were “named” based on their respective score patterns on the 10 items. The score pattern of the 10 items for T1 data and T2 data were shown in Figures 1 and 2, respectively.

The three subgroups of T1 were named the *well‐adjusted group*, the *conduct problem group* and the *attention disorder group*. In the *well‐adjusted group*, the respondents generally had relatively low scores on all the 10 items, this profile group was the largest of the three profiles groups at both time points, and showed some reduction from T1 to T2 (66.3% at T1 and 59.0% at T2, respectively). For the *conduct problem group*, the respondents had the score pattern of relatively higher scores on the conduct problem subscale “following the command” (SDQ3), “having disputes with others” (SDQ5), “lying often” (SDQ7), and “taking others' things” (SDQ9). The proportion of this profile group showed some increase from T1 (14.1%) to T2 (19.3%). For the *Attention disorder group*, the respondents had the score pattern of higher scores on “failure to keep quiet for a long time” (SDQ1), “often feeling restless or impatient” (SDQ4), “thinking before doing something” (SDQ8), and “good concentration” (SDQ10). The proportion of this subgroup increased slightly from T1 (19.6%) to T2 (21.8%). The three subgroups ofT2 scored in a similar manner to T1, again named the *well‐adjusted group, attention disorder group*, and *conduct problem group*.

It is worth noting that the fifth question in the scale “I feel very angry often temper”, *attention disorder group* and *conduct problem group* subjects in this item all score is higher, and *well‐adjusted group* in this low score, this is the previous studies using externalized problem behavior score or dimension total score will be directly classified ignored content. This may suggest that in early adolescence, regardless of their status in any subcategory group, The most basic and common feature is the frequent anger (Brotman et al. 2017).

### 3.4 Unconditional Latent Transition Model

The results of the unconditional Latent Transition Analysis are as shown in Table 3. The diagonal of the transition matrix indicates the probability that individuals would remain in the same latent profile group from T1 to T2. Individuals in the two latent profile groups of *well‐adjusted group* and *attention disorder group* had relatively high probabilities (0.67 and 0.65, respectively) of remaining in the same profile group across T1 and T2. Individuals in the *conduct problem group*, however, had a lower probability of remaining in the same latent profile group at T2 (0.52). Among adolescents who made the transition, those who were in the *conduct problem group* at T1 were more likely to transition to the *well‐adjusted group* over time (40% probability of transition).

**TABLE 3:** Transition probability of being in the same or different profile groups from T1 to T2 (*N* = 752).

Subgroup at T1 (2017)	Subgroup at T2 (2019)		
	A	B	C
A—Well‐adjusted group	**0.67**	0.11	0.22
B—Attention disorder group	0.13	**0.65**	0.22
C—Conduct problem group	0.40	0.08	**0.52**

*Note*: The bold values indicate the probability that individuals would remain in the same latent profile group from T1 to T2.

### 3.5 Latent Transition Model With Covariates

To understand the potential effects, or lack thereof, of the demographic and family background variables (gender, parents education, family function, and parental marriage quality) on the longitudinal transition across the three profile groups in this sample of early adolescents in poverty over a 14‐months span, we conducted conditional latent transition analysis to examine how the demographic and family background variables could have influenced the transitioning across the three profile groups from T1 to T2. In the analyses for transitioning across different profiles of groups from T1 to T2, those who remained in the same profile group at both times (T1 and T2) were used as the reference group. This means that the OR from this latent transition analysis conceptually quantified the likelihood of individuals changing into a different profile group at T2 relative to the likelihood of remaining in the same profile group at both times. OR > 1 of a predictor would suggest that, relative to those with no change of profile status from T1 to T2, the predictor would result in a higher likelihood for transition from one profile group at T1 to another profile group at T2. Conversely, OR < 1 would suggest a lower likelihood of such a transition.

According to Table 4, compared to the proportion in the *well‐adjusted group*, boys had a lower proportion in the *attention disorder group*, 0.63 times the proportion in the *well‐adjusted group*, and girls had a higher proportion in the *conduct problem group*, 1.13 times the proportion in the *well‐adjusted group*. The incidence ratios for the influences of parent education level, family function, and parental marital quality were all less than 1, suggesting that all of these influences may reduce the level of externalizing problem behavior, but to different extents. Among them, subjects with high parental education, good family function, and high parental marital quality had a lower probability of belonging to the *conduct problem group*, 0.64, 0.84, and 0.79 times that of the *well‐adjusted group*, respectively. Poor children with high parental marital quality were less likely to be in the *attention disorder group*.

**TABLE 4:** Odds ratios of latent state probabilities at T1 under the influence of covariates (*N* = 752).

Influence factor	Conduct problem group	Attention disorder group	Well‐adjusted group	*p*
Gender^a^	1.13**	0.63**	REF	< 0.05
Parent education level^b^	0.64**	1.06	REF	< 0.05
Family function	0.84**	0.97	REF	< 0.01
Parental marital quality	0.79**	0.85*	REF	< 0.01

^a^ Gender (1 = boy, 2 = girl) and parents' education level.

^b^ Dummy variables.

The results presented in Table 5 indicated that, with regard to the demographic variable Gender, among those in the *attention disorder group* at T1, girls were much more likely (OR = 2.63, *p* < 0.01) to transition into the *well‐adjusted group* and the *conduct problem group* (OR = 1.29, *p* < 0.05) at T2. Among those in the *well‐adjusted group* at T1, girls were somewhat less likely than boys to transition into the *attention disorder group* at T2 (OR = 0.81, *p* < 0.05). Among those in the *conduct problem group* at T1, girls were much less likely to transition into the *conduct problem group* (OR = 0.55, *p* < 0.01) than boys at T2.

**TABLE 5:** Odds ratios of the covariates for transition probability from T1 to T2 (*N* = 752).

Predictive variable	T1 subgroups	T2 subgroups		
		I	II	III
Gender^a^	I—Attention disorder group	REF	2.63**	1.29*
	II—Well‐adjusted group	0.81*	REF	0.90
	III—Conduct problem group	0.95	0.55**	REF
Parent education level^b^	I—Attention disorder group	REF	1.37**	0.69**
	II—Well‐adjusted group	0.98	REF	0.80*
	III—Conduct problem group	1.16*	1.58**	REF
Family function	I—Attention disorder group	REF	1.16*	1.04
	II—Well‐adjusted group	0.71**	REF	0.92
	III—Conduct problem group	1.25**	1.34**	REF
Parental marital quality	I—Attention disorder group	REF	0.87*	0.98
	II—Well‐adjusted group	0.97	REF	0.47**
	III—Conduct problem group	1.08	1.61**	REF

^a^ Gender (1 = boy, 2 = girl) and parents' education level.

^b^ Dummy variables.

With regard to parent education level, among the children in the *attention disorder group* at T1, those with a higher parent education level were more likely to transition into the *well‐adjusted group* (OR = 1.37, *p* < 0.01) at T2 than to remain in the same. More problematic, *attention disorder group*, and they were much less likely to transition into the more problematic *conduct problem group* at T2 (OR = 0.69, *p* < 0.01). Among those in the *well‐adjusted group* at T1, those with a higher parent education level were somewhat less likely to transition into the more problematic *conduct problem group* at T2 (OR = 0.80, *p* < 0.05). Among those in the *conduct problem group* at T1, those with a higher parent education level were somewhat more likely to transition into the *attention disorder group* at T2 (OR = 1.16, *p* < 0.05), and they were much more likely to transition into the *well‐adjusted group* at T2 (OR = 1.58, *p* < 0.01). All these findings indicated the positive role of parent education in facilitating the transition of the early adolescents in poverty from the more problematic behavior group (i.e., *conduct problem group*) to the less problematic behavior group (e.g., *attention disorder group* and *well‐adjusted group*).

With regard to family function as a predictor, the findings were similar to those of parent education level, as family function showed a positive role in facilitating the transition from a more problematic behavior group at T1 to a less problematic group at T2. More specifically, among those in the *attention disorder group* at T1, those from families with better family function were somewhat more likely to transition into the *well‐adjusted group* (OR = 1.16, *p* < 0.05) than remaining in the *attention disorder group* at T2. Similarly, among those in the *conduct problem group* at T1, those from families with better family function were much more likely to transition either into the less problematic *attention disorder group* (OR = 1.25, *p* < 0.01) at T2 or into the *well‐adjusted group* (OR = 1.34, *p* < 0.01) at T2 than to remain in the more problematic *conduct problem group*. Among those in the *well‐adjusted group* at T1, those from families with better family function were much less likely to transition into the problematic *attention disorder group* at T2 (OR = 0.71, *p* < 0.01). In short, family function appeared to have played a positive role in facilitating the adolescents in poverty to transition into less problematic groups of externalizing problem behaviors over the 14‐months period.

The final predictor in the model, parent marital quality, showed a similar positive role as the previous two family background variables in facilitating the early adolescents' transition from a more problematic group to a less problematic group (e.g., from *attention disorder group* to *well‐adjusted group*, OR = 0.87, *p* < 0.05; from *conduct problem group* to w*ell‐adjusted group*, OR = 1.61, *p* < 0.01), or mitigating against the transition from a less problematic group to a more problematic group (e.g., from the *well‐adjusted group* to *conduct problem group*, OR = 0.47, *p* < 0.01).

## 4 Discussion

In this study, the externalizing problem behaviors of early adolescents from poverty‐stricken families were examined and classified into three profiles (*well‐adjusted*, *attention disorder group*, and *conduct problem group*). The study further examined the transition from one profile group into another profile group over 14 months. The influences of gender and family background variables (gender, parent education level, family function, parent marital quality) on the longitudinal transition patterns were examined in latent transition analysis.

### 4.1 Latent Profiles of Externalizing Problem Behavior Patterns

This study explored subgroups of differentiation in early adolescent externalizing behavior problems using latent profile analysis. From the *Strengths and Difficulties Questionnaire* (SDQ; Goodman et al. 1998), based on the responses to the 10 items on the two sub‐scales (conduct problems, hyperactivity disorder) for measuring externalizing problem behaviors of children (Roman et al. 2016), latent profile analysis (LPA) suggested three latent profiles: *well‐adjusted*, *attention disorder*, and *conduct disorder*. The *attention disorder group* was characterized by relatively high scores on the set of items related to attention (e.g., “failure to keep quiet for a long time,” “often feeling restless or impatient”), but relatively low scores on the set of items related to conduct (e.g., “having disputes with others,” “lying often,” and “taking others' things”). The *conduct problem group*, on the other hand, showed the opposite score pattern (i.e., high scores on conduct items, and relatively low scores on attention items). The *well‐adjusted group* had relatively low scores on both sets of items, and this group was the largest (66% at T1) in the sample.

The *attention disorder group* had a higher proportion in the sample (19.6% at T1) than that of the *conduct problem group* (14.1% at T1), while this gap decreased at T2 (21.8% in the *attention disorder group* and 19.3% in the *conduct problem group*). This was consistent with previous studies: early externalizing problem behaviors tended to be related to hyperactivity, and conduct behavior problems tended to emerge later. That is, in early adolescence, more children in poverty could have attention disorder than those with conduct problems (Flouri et al. 2018). Therefore, parents and teachers should pay extra attention to attention problems in early adolescence.

### 4.2 Longitudinal Transition Across Latent Profiles of Externalizing Problem Behaviors

The findings of Donker et al. (2003) indicated that early adolescents had more externalizing problem behaviors, and they were less stable than their younger or older peers. Therefore, this study explored the transformation of externalizing behavior problems in early adolescence. The findings about the transitional probabilities (Table 3) across different profile groups from T1 to T2 indicated that, for the three profile groups (*well‐adjusted group, attention disorder group* and *conduct problem group*), the probabilities of remaining in the same group over 14‐months span (i.e., from T1 to T2) were relatively high (0.67, 0.65, and 0.52, respectively). Of these, individuals in the behavior problem group had the lowest stability. Development with time tends to gradually shift to a low externalizing problem group (0.52). The stability differences between the *attention disorder group* and the *conduct problem group* were not discussed in the research literature before. However, such differences did make intuitive sense, although future research is needed to further examine this issue. In school settings, attention disorder, including lack of concentration in class and poor academic performance, typically affects an individual him/herself. Parents and teachers may attribute such behaviors to individual learning styles and/or abilities; as a result, attention disorder may be more easily overlooked and ignored (Sorkkila et al. 2017). Conduct problems, including fighting, stealing, smoking, drinking, and other behaviors, on the other hand, usually affect others; as such, these behaviors could attract more attention from parents and teachers, and would more easily lead to interventions and actions intended to correct such externalizing problem behaviors (Yun et al. 2016), thus leading to behavior change by some early adolescents over time.

More importantly, a considerable proportion in this *conduct problem group* tended to transition into the *well‐adjusted group* (OR = 0.40) during the 14 months span, suggesting that some early adolescents from poverty‐stricken families with conduct problems would gradually change for the better. This also supports the positive adolescent development perspective, which suggests that adolescents themselves harbor the potential for positive development (Damon 2004) and are intrinsically motivated to move in the good direction even in adverse situations (Moore et al. 2019). Therefore, it is important to take preventive measures to keep more adolescents on track for good adjustment during the critical period of adolescent divergence, and to provide more targeted guidance or interventions for different adolescent subgroups.

### 4.3 Factors Influencing Behavior Change in Externalizing Problems

In research on factors that could influence the externalizing problem behaviors of children, cross‐sectional design has been dominant. This study used a longitudinal design to examine the influence of gender and family background variables on the development of externalizing problem behaviors of early adolescents in poverty over 14 months. The present study found that gender influences the transition pattern of externalizing behavior problems, i.e., boys in the behavior problem group were more likely to transition to the normal behavior group, whereas girls in the attention disorder group were more likely to transition to the normal behavior group. As discussed in previous literature, boys' bodies are physiologically more dynamic, causing boys to exhibit more externalizing problem behaviors such as vandalism and physical aggression; the social environment encourages girls to be submissive and behave gently (Brotman et al. 2017). Therefore, the occurrence of conduct problems among boys was considerably higher than that among girls, while the occurrence of attention disorder among girls was typically higher than that among boys (Van Heel et al. 2020). As children entered adolescence and were slowly separated from families integrated into school. The school will intervene to correct the externalizing problem behaviors that are highlighted, the boys' aggressive behaviors (i.e., conduct problem behaviors) would gradually ease, and the gender difference in conduct problem behaviors would become smaller (Clarizio 1997).

The more important findings of this study were related to the family background variables, about the roles that these family background variables played in the transition of these early adolescents from poor families across different profile groups over 14 months. More specifically, the empirical findings from the latent transition analysis indicated that the three family variables (parents education, family function, and parents' marriage quality) all played positive roles in facilitating the adolescents' transition from *conduct problem group* into *well‐adjusted group* and in mitigating against the adolescents' transition from less problematic or normal behavior group into a group with more problematic behaviors.

In this study, the higher the educational level of the parents in the family, the lower the level of externalizing problem behavior in the poor children. Family function has a negative effect on the initiation level of early adolescence in poor children, suggesting that children from families with better family functioning have a lower initial level of externalizing problem behavior. This is consistent with the results of previous studies (Coe et al. 2018). Children growing up in better functioning families were more likely to have better psychosocial adaptations and fewer externalizing problems (Linville et al. 2010). Children from families with better marriage quality have a lower initial level of externalizing problem behavior. This result is in line with the second pathway in the family stress model: poverty situation‐parental stress‐parental marriage quality‐child behavior problem (Masarik and Conger 2017). Davies and Cummings's emotional safety hypothesis also holds that parental conflict can affect children's emotional security by influencing children's problematic behavior (Hentges et al. 2015). When the marriage quality of the parents is low, there will be more conflict behaviors in front of their children, and the children may imperceptibly acquire the high conflict relationship mode of their parents, leading to the high level of externalizing problem behavior.

Previous studies showed that children from families in adverse situations over extended periods of time (e.g., marriage pressure, financial pressure, negative parenting, etc.) were more likely to have more externalizing problem behaviors (Bartels et al. 2004). But the positive family environment plays a positive role in reducing the severity of the externalizing problem behaviors of the early adolescents in poverty has not been examined in such or similar longitudinal contexts before. Such findings were conceptually consistent with some prevailing views concerning children and adolescents, especially those from poor family backgrounds, as family was often the microsystem with the strongest influence on the development of children and adolescents (Brock and Kochanska 2016). As such, early adolescents in poverty could have their externalizing problem behaviors shaped and affected by some relevant family factors (Masarik and Conger 2017), such as those examined in this study. Therefore, the findings from the latent transition analysis further highlight the importance of a good family environment in reducing the externalizing problem behaviors among early adolescents in poverty.

### 4.4 Practical Implications and Study Limitations

The results of this study provide some useful insights about externalizing problem behaviors of early adolescents from poor families. First, attention disorder among the early adolescents in poverty appeared to be stable, and may easily be overlooked by parents and teachers. However, a higher level of attention disorder could suggest a higher risk of attention deficit hyperactivity disorder (ADHD) (Eichelberger et al. 2016; Kaori et al. 2020). Thus, more attention to children and adolescents having attention disorder would be encouraged. Second, factors that could reduce the externalizing problem behaviors of adolescents in poverty should be considered. Adolescents in poverty with high‐level externalizing problem behaviors often had anger as a result of disadvantaged family situations. Professional support and help could be needed in guiding their parents in having more and better communication with their children in relieving such anger. Such active and effective communication could facilitate their children's transition to having less problematic externalizing behaviors (Van Heel et al. 2019). Third, the gender differences in externalizing problem behaviors of the early adolescents in poverty should also receive our attention. More attention disorder problems among boys and more behavioral problems among girls suggest that more targeted help should be provided to them. Most importantly, we found that family background variables were protective factors against the transition of individuals from the *well‐adjusted group* to the severe *conduct problem group* in early adolescence and also facilitated the positive transition of individuals from the *conduct problem group* to the *well‐adjusted group*.

This study has some limitations. First, for externalizing problem behaviors in the study, we used the self‐report data from the adolescents from poor families. As known in research literature, self‐report data from adolescents could have some validity issues (e.g., the social expectation effect, intentional hiding of some problem behaviors, or only reporting more serious problem behaviors; Butovskaya et al. 2007). In future studies, multiple data methods may be utilized for triangulation, in order to avoid some pitfalls associated with self‐report data and to enhance data validity and quality. Second, although a longitudinal design was used in this study, the time frame of 14 months could be too short, thus limiting our understanding of the transition of externalizing problem behaviors among the adolescents in poverty. Future studies may consider a longitudinal design with a longer time frame and with more than two data collection points, so that we can develop a better understanding of the transitions and changes of externalizing problem behavior patterns among early adolescents from poor families. Third, only the conduct problems and hyperactivity disorder subscales of the SDQ scale were used in this study. Therefore, great care should be taken in generalizing the results of this study to other more severe externalizing problems.

## 5 Conclusion

1. There are three potential profiles of externalizing problem behaviors in early adolescence: *well‐adjusted group*, *attention disorder group*, and *conduct problem group*.
2. The conduct disorder group showed a tendency to transition to the well‐adjusted group.
3. Supportive family functioning is a positive factor in mitigating externalizing problem behaviors in adolescents from poverty‐stricken families during the transition to adolescence.

## Ethics Statement

The study was reviewed and approved by the Ethics Committees of the Institute of Education, Hunan University of Science & Technology. Written informed consent to participate in this study was provided by the participants' legal guardian/next of kin.

## Conflicts of Interest

The authors declare no conflicts of interest.
